# The electronic cigarette: the new cigarette of the 21st century?*

**DOI:** 10.1590/S1806-37132014000500013

**Published:** 2014

**Authors:** Marli Maria Knorst, Igor Gorski Benedetto, Mariana Costa Hoffmeister, Marcelo Basso Gazzana

**Affiliations:** Federal University of Rio Grande do Sul School of Medicine; and Physician. Department of Pulmonology, Porto Alegre Hospital de Clínicas, Porto Alegre, Brazil; Graduate Program in Pulmonology, Federal University of Rio Grande do Sul, Porto Alegre, Brazil; Federal University of Rio Grande do Sul School of Medicine, Porto Alegre, Brazil; Department of Pulmonology, Porto Alegre Hospital de Clínicas; and Department of Pulmonology and Thoracic Surgery, Moinhos de Vento Hospital, Porto Alegre, Brazil

**Keywords:** Smoking, Tobacco Products, Nicotine

## Abstract

The electronic nicotine delivery system, also known as the electronic cigarette, is generating considerable controversy, not only in the general population but also among health professionals. Smokers the world over have been increasingly using electronic cigarettes as an aid to smoking cessation and as a substitute for conventional cigarettes. There are few available data regarding the safety of electronic cigarettes. There is as yet no evidence that electronic cigarettes are effective in treating nicotine addiction. Some smokers have reported using electronic cigarettes for over a year, often combined with conventional cigarettes, thus prolonging nicotine addiction. In addition, the increasing use of electronic cigarettes by adolescents is a cause for concern. The objective of this study was to describe electronic cigarettes and their components, as well as to review the literature regarding their safety; their impact on smoking initiation and smoking cessation; and regulatory issues related to their use.

## Introduction

Smoking is a major public health problem worldwide and is considered by the World Health Organization (WHO) to be one of the leading causes of preventable death.^(^ 1 ^)^ In Brazil, approximately 220,000 tobacco-related deaths occur each year. ^(^ 2 ^)^ Nevertheless, 16.1% of all Brazilian adults are smokers; of those, 17 million are male and 12.5 million are female.^(^ 3 ^)^ Concerns regarding the morbidity and mortality associated with smoking led to the WHO Framework Convention on Tobacco Control (FCTC), which was implemented on February 27, 2005 and was ratified by 177 countries, including Brazil. ^(^ 4 ^)^ Among the guidelines for implementation of the WHO FCTC are the promotion of smoke-free environments and the implementation of smoking cessation projects. In accordance with the WHO FCTC, the Brazilian National Ministry of Health developed a smoking cessation program based on cognitive-behavioral therapy and pharmacological treatment, the program being implemented in the Brazilian Unified Health Care System.^(^ 5 ^)^

Studies of smokers have shown that many would not smoke if they had their time again^(^ 6 ^)^ and that 60-70% wanted to quit smoking.^(^ 7 ^)^ However, without assistance, most of those who attempt to quit smoking relapse, and only 4% remain abstinent at one year.^(^ 8 ^)^ One of the most important factors that make smoking cessation more difficult is nicotine dependence. In this context, the electronic cigarette (EC, also known as e-cigarette) has emerged as a form of nicotine replacement therapy. The EC was developed by Chinese pharmacist Hon Lik and was patented in 2003.^(^ 9 ^)^ Although there is a lack of data on their efficacy and safety, ECs are widely available for purchase on the Internet, as well as being sold directly to consumers in various countries. 

Currently, more than 2,500 brands of ECs are sold worldwide.^(^ 10 ^)^ Several of these brands have been acquired by the tobacco industry. In the USA, the price of an EC ranges from U$ 29.95 to U$ 149.95, and the price of an EC cartridge ranges from U$ 9.95 to U$ 19.95.^(^ 11 ^)^ In Brazil, the National Health Surveillance Agency prohibited the commercialization, importation, and advertising of ECs in 2009.^(^ 12 ^)^ Nevertheless, smokers have access to ECs and often seek advice from pulmonologists regarding the recommendations for use and efficacy of ECs. The objective of the present study was to describe ECs and their components, as well as to review the available evidence regarding their role in smoking cessation; their impact on smoking initiation; their safety; and ethical and regulatory issues related to their use. 

## Characteristics of ECs

**Figure 1:**
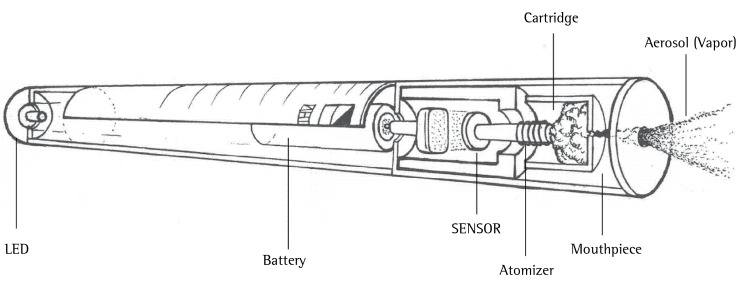
Electronic cigarette components. LED: light-emitting diode.

The EC is an electronic device that provides users with an aerosol containing nicotine and other additives. The three main components of an EC are a battery, an atomizer, and a cartridge containing nicotine (Figure 1). Nicotine-free ECs are commercialized in some countries.^(^ 13 ^)^ Some ECs have, on one end, a light-emitting diode that lights up when the device is used, thus reminding users that the cigarette is lit. Most electronic nicotine delivery systems mimic traditional forms of tobacco use, i.e., cigarettes, cigars, and pipes; less commonly, electronic nicotine delivery systems resemble daily use objects such as a pen or a USB flash drive, being primarily used by individuals who want to smoke without attracting attention.^(^ 14 ^)^

Although the components of an EC cartridge vary according to the brand, EC cartridges usually contain nicotine and a component aimed at producing an aerosol (e.g., propylene glycol or glycerol diluted in water). The nicotine concentration in an EC cartridge can vary and might not correspond to that described by the manufacturer.^(^ 15 ^,^ 16 ^)^ Some brands of ECs contain flavorings such as fruit extract, vanilla, mint, coffee, and chocolate, which make ECs more attractive, especially to adolescents. Several potentially harmful substances, such as formaldehyde, acetaldehyde, acrolein, volatile organic compounds, heavy metals, and tobacco-specific nitrosamines, have been identified in nicotine cartridges.^(^ 15 ^,^ 17 ^)^

When users draw air through an EC, a sensor detects air flow and heats the liquid in the cartridge, which is vaporized. The aerosol delivers nicotine to EC users, and part of the EC aerosol is released into ambient air when users exhale. The temperature of the EC aerosol ranges from 40°C to 65°C. According to the manufacturers, a single EC cartridge can yield 10-250 puffs, corresponding to 5-30 conventional cigarettes (depending on the brand).^(^ 18 ^)^ Second- and third-generation ECs have recently been developed; they have batteries and atomizers that are more powerful and can deliver higher doses of nicotine, thus increasing the risk of addiction.^(^ 19 ^)^

## Prevalence of EC use in adults

Although there is a lack of evidence that the EC is effective in smoking cessation, the interest in ECs is increasing, as is the number of EC users worldwide, most of whom are adult smokers.^(^ 20 ^-^ 22 ^)^ Users of ECs identify themselves as "vapers". In the USA, a survey of more than 10,000 adults showed that knowledge of the existence of ECs doubled between 2009 and 2010 (from 16.4% to 32.2%), and the use of ECs nearly quadrupled (from 0.6% in 2009 to 2.7% in 2010).^(^ 23 ^)^ Among active smokers, 11.4% reported having used ECs, and 4.1% reported having used ECs in the past 30 days.^(^ 24 ^)^ In Great Britain, the proportion of regular EC users increased from 2.7% in 2010 to 6.7% in 2012.^(^ 25 ^)^ Data collected between 2010 and 2011 from 5,939 individuals in four countries (the USA, the UK, Canada, and Australia) showed that approximately half of the interviewees (46.6%) were aware of the existence of ECs. However, the proportion of individuals that were aware of the existence of ECs varied significantly among the countries studied, being higher in the USA (73.4%) and the UK (54.4%), where EC use is allowed, and lower in Canada (39.5%) and Australia (20.0%), where EC use has been banned. The rate of experimentation was 7.6% (being 16.3% among those who were aware of ECs), and the rate of current use was 3%; the proportion of current EC users did not vary among the countries studied (p = 0.114).^(^ 26 ^)^

An online forum on smoking cessation and ECs held in England and France in 2010 brought together 3,587 participants (former smokers, 70%; males, 61%; mean age, 41 years). Nicotine-containing ECs were used by 97% of the participants, being used for approximately five months by former smokers. Most reported that ECs helped them quit or reduce smoking (96%). Reasons for EC use included the perception that ECs were less toxic than conventional cigarettes (87%), a reduction in tobacco craving (79%), a reduction in withdrawal symptoms (77%), the fact that ECs were less expensive than tobacco (57%), and control of situations in which smoking was prohibited (39%).^(^ 27 ^)^ One limitation of the study was the selection of a convenience sample. An online survey of 81 smokers showed the pattern of EC use among regular EC users. The median duration of EC use was 100 days, and the median number of puffs/day was 175.^(^ 28 ^)^

A recent systematic review of 49 studies showed that knowledge of the existence of ECs increased from 16% in 2009 to 58% in 2011 and that the use of ECs increased from 1% to 6% in the same period.^(^ 22 ^)^

## Adolescent exposure to ECs

Children and adolescents in several countries are aware of and have access to ECs. An online survey of 228 American male adolescents showed that 67% were aware of ECs, although less than 1% reported having experimented with ECs.^(^ 29 ^)^ In a study of 444 Korean adolescents, 10.2% reported having seen or heard of ECs and 0.5% reported having used ECs. Contact with ECs was through the Internet in 46% of cases; friends, in 27.9%; television, in 11.0%; books, in 9.3%; and others, in 5.4%. Male adolescents were 6.3 times more likely to use ECs than were female adolescents, and adolescents with smokers in the family were 3.4 times more likely to use ECs than were those without.^(^ 30 ^)^

The prevalence of EC use and conventional cigarette smoking among US adolescents in grades 6th-12th in the 2011-2012 period was assessed in a cross-sectional study (National Youth Tobacco Survey).^(^ 31 ^)^ The results showed that EC experimentation and recent EC use nearly doubled in the study period. The use of ECs increased from 3.3% to 6.8% (p < 0.05), the use of conventional cigarettes increased from 1.1% to 2.1% (p < 0.05), and the use of both ECs and conventional cigarettes increased from 0.8% to 1.6% (p = 0.05) in the study period. There were no differences between the adolescents in the 6th-8th grade and those in the 9th-12th grade regarding the aforementioned increases. The study also showed that, in 2012, 9.3% of all EC experimenters reported never smoking conventional cigarettes and that 76% of all regular EC users reported smoking conventional cigarettes regularly.^(^ 32 ^)^

A study conducted in eight schools in North Carolina, USA, and involving 4,444 adolescents in the 11-19 year age bracket showed that 4.9% reported the use of ECs, 1.5% having reported the use of ECs in the past month. Although EC use was more common among conventional cigarette smokers, 12% of all EC users had never smoked conventional cigarettes.^(^ 33 ^)^

The Internet advertising and online marketing of ECs, even in countries where ECs have been banned, can encourage EC use and allow adolescents to have access to ECs. In addition, data from the aforementioned studies^(^ 29 ^-^ 33 ^)^ suggest that EC experimentation induces continued use of conventional cigarettes during adolescence. Therefore, measures aimed at reducing the appeal of EC use and prohibiting the sale of ECs to adolescents are essential to minimize the risk of tobacco and EC use. 

## Safety of EC use

The safety of electronic nicotine delivery systems has yet to be scientifically demonstrated, and health risks of EC use have yet to be determined. Most EC safety issues are due to the lack of appropriate regulation and inconsistent quality control. Because of the lack of regulation and surveillance, the quality of ECs, the amount of nicotine delivered, and the components of EC cartridges vary widely across brands.^(^ 15 ^)^ Therefore, EC users cannot know the exact composition of the product that they are using. 

Adverse effects of EC use might be due to variations in the nicotine content of EC cartridges. According to the manufacturers, the nicotine content of an EC cartridge can range from 6 mg to 24 mg; however, nicotine concentrations as high as 100 mg per cartridge have been detected. Therefore, the risk of poisoning should be taken into consideration. When nicotine is inhaled, is ingested, or comes into contact with the skin, it can be dangerous to the health of vulnerable individuals, such as children, youth, pregnant women, lactating women, individuals with heart disease, and the elderly. Large quantities of nicotine (i.e., 0.5-1.0 mg of nicotine per kg of body weight) can be lethal, and it is therefore recommended that ECs, EC cartridges, and refills be kept out of the reach of children.^(^ 14 ^,^ 16 ^)^

The health risks of EC use might also be associated with the various substances found in EC refill cartridges. One such substance is propylene glycol, in which nicotine is suspended and which is used in order to generate the EC aerosol. Data on the harmful effects of inhaling propylene glycol are scarce. Eye irritation and upper airway irritation, as well as cough and mild airway obstruction, have been reported to occur in individuals without asthma after short-term exposure to the propylene glycol mist created by an artificial smoke generator.^(^ 34 ^)^

Other potentially harmful substances, including irritants and toxins such as diethylene glycol, formaldehyde, acetaldehyde, and acrolein, were detected in some EC brands.^(^ 17 ^)^ Nitrosamines, which are well-recognized carcinogens,^(^ 35 ^)^ as well as tobacco-specific impurities, were found in low concentrations in two brands of ECs.^(^ 17 ^)^ The EC might contain flavorings, which are added to EC cartridges in order to make ECs more palatable. Although these substances are routinely used food flavorings, the effects of inhaling them are unknown. 

By the first quarter of 2012, the US Food and Drug Administration had received 49 reports of adverse events related to the use of ECs. Of those adverse events, 8 were considered serious, including pneumonia and chest pain; the remaining events were characterized as mild and included headache and cough.^(^ 36 ^)^ Among other symptoms, headache, mouth and throat irritation, salivation, sweating, weakness, palpitations, nausea, vomiting, and diarrhea were reported in a study evaluating acute adverse effects 2.5 h after EC use. However, all of the aforementioned effects were mild.^(^ 37 ^)^ In three prospective studies in which smokers used ECs for 6 or 12 months, no serious adverse events were observed, and the main complaints were cough, headache, and mouth and throat irritation. The symptoms resolved or subsided with continued EC use.^(^ 38 ^-^ 41 ^)^

In vivo and in vitro studies have evaluated the impact of EC aerosol on blood cells and the cytotoxic effect of EC aerosol on myocardial cells. In an in vivo study, one group of authors found that neither active EC use nor passive exposure to EC aerosol for 30 min affected leukocyte, lymphocyte, or granulocyte counts.^(^ 42 ^)^ In an in vitro study, another group of authors evaluated the cytotoxic effects that the aerosol produced by 20 EC brands had on myocardial cells in culture.^(^ 43 ^)^ Although some samples were found to have cytotoxic effects on myocardial cells, the cytotoxicity of EC aerosol was found to be lower than was that of conventional cigarette smoke.^(^ 43 ^)^

The effects of EC use on lung function have been studied, although only after acute exposure. Neither active EC use for a few minutes (in smokers) nor passive exposure to EC aerosol for 1 h (in nonsmokers) had any effect on FEV_1_.^(^ 44 ^)^ In contrast, EC use for 5 min increased airway resistance and reduced the fraction of exhaled nitric oxide in adult smokers without comorbidities.^(^ 45 ^)^ Increased airway resistance can precede changes in PEF and FEV_1_ in experimentally induced airflow obstruction.^(^ 46 ^)^ Nitric oxide is recognized to play a role in the pathophysiology of smoking-related airway disease; in addition, nitric oxide is related to eosinophilic inflammation and bronchial hyperreactivity, as well as being a marker of oxidative stress.^(^ 47 ^)^ Taken together, the aforementioned findings suggest that short-term EC use induces pulmonary changes. Long-term effects of EC use on lung function have yet to be studied. 

Given that ECs do not generate the smoke that is associated with the combustion of tobacco, EC use is generally considered safer than tobacco use. This "relative safety" can be appealing to users; however, the chemicals used in ECs have yet to be fully disclosed, and data on the environmental pollution generated by the use of ECs in enclosed spaces are scarce. One study evaluated the air quality in a room in which 9 individuals used ECs. The results showed substantial quantities of 1,2-propanediol, glycerin, and nicotine, as well as high concentrations of particulate matter of 2.5 mm in diameter, together with a 20% increase in the levels of polycyclic aromatic hydrocarbons and an increase in the levels of aluminum and total particulate matter. The concentration of exhaled nitric oxide increased in 7 of the 9 individuals studied.^(^ 48 ^)^ In addition, there are currently no data on the safety of long-term EC use.^(^ 14 ^)^

## Efficacy of ECs in smoking cessation

There are currently few available data on ECs. According to the WHO, there is no scientific evidence for the use of ECs as a substitute for conventional cigarettes or as an aid to smoking cessation. In addition, unlike approved nicotine replacement therapies (e.g., nicotine patches, gum, and lozenges), ECs deliver nicotine directly to the lungs and therefore must be further studied.^(^ 14 ^)^

A comparison of different EC brands showed varying concentrations of nicotine in the cartridges, as well as varying aerosol content and inconsistent nicotine delivery. After EC use, plasma nicotine levels remained unchanged in all of the patients in two studies^(^ 49 ^,^ 50 ^)^ and in approximately one third of the cases in another study.^(^ 51 ^)^ However, in regular EC users, plasma nicotine levels can increase,^(^ 52 ^)^ although serum cotinine levels have been found to vary widely among individuals.^(^ 37 ^)^

Several studies have evaluated the impact of ECs on the urge to smoke and on cravings. In a randomized crossover study sponsored by an EC manufacturer, an electronic nicotine delivery device containing 16 mg of nicotine was found to be more effective than placebo in relieving morning withdrawal symptoms following overnight abstinence in 40 smokers. The effects of the electronic nicotine delivery device on nicotine withdrawal symptoms were comparable to those of a nicotine inhaler but lower than those of conventional cigarettes.^(^ 51 ^)^ One group of authors^(^ 53 ^)^ studied the effects of an EC on the urge to smoke, nicotine withdrawal symptoms, and cognition in 86 patients randomly divided into three groups: a) 18-mg nicotine EC (the nicotine group); b) nicotine-free EC (the placebo group); and c) just hold the EC (the just hold group). Within 20 min after EC use, the urge to smoke and nicotine withdrawal symptoms were significantly reduced in the nicotine and placebo groups in comparison with the just hold group. Regarding the reduction in the urge to smoke, the nicotine EC was significantly superior to placebo in males but not in females. However, memory test results were significantly better in the nicotine group. In another crossover study, an acute exposure protocol consisting of 10 sequential puffs, 30 s apart, was used in order to compare four experimental conditions: an EC brand with a 16-mg nicotine cartridge; another EC brand with a 16-mg nicotine cartridge; own brand cigarettes; and an unlit cigarette (placebo). When compared with placebo, one of the EC brands tested reduced craving; however, the effect was lower than was that of conventional cigarettes.^(^ 50 ^)^

**Chart 1:**
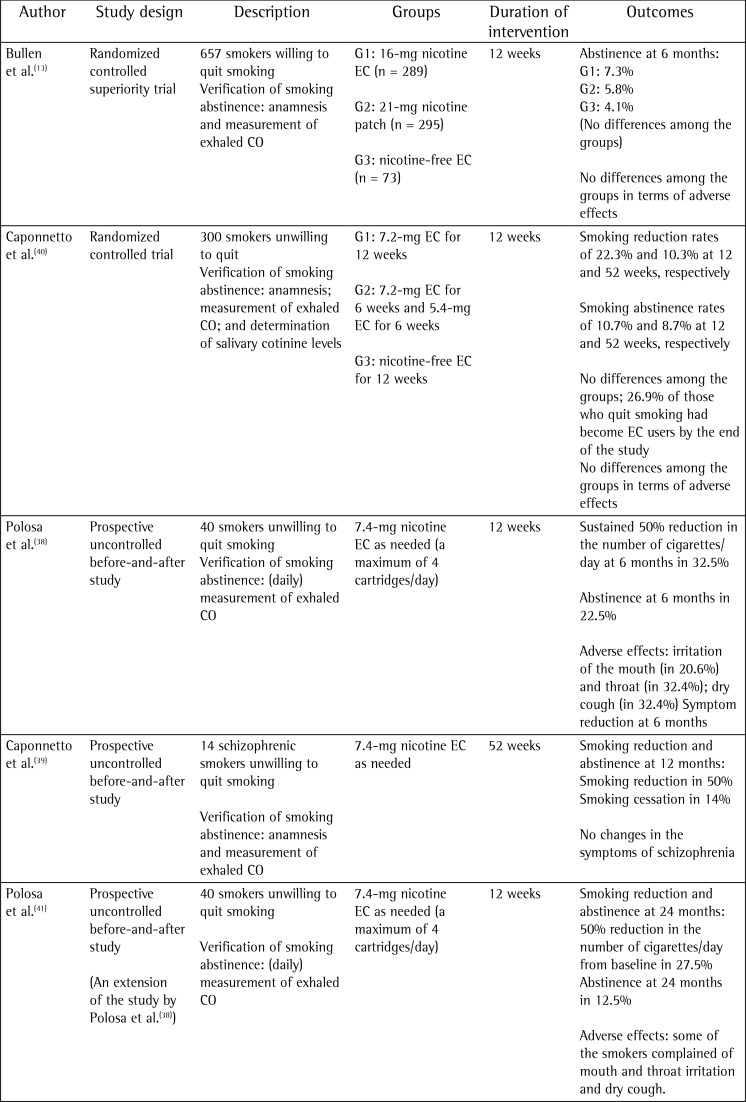
Clinical studies evaluating the effects of electronic cigarettes on smoking reduction and smoking cessation. CO: carbon monoxide; EC: electronic cigarette; and G1, G2, and G3: groups 1, 2, and 3.

Few studies have evaluated the effects of EC use on smoking reduction and cessation within 6-24 months. Two randomized controlled clinical trials and three prospective before-and-after studies are described in Chart 1. One of the clinical trials^(^ 13 ^)^ compared a 16-mg nicotine EC with 21-mg nicotine patches and a nicotine-free EC, whereas the other^(^ 40 ^)^ compared a 7.2-mg nicotine EC with a nicotine-free EC. Both trials lasted 12 weeks, and neither found significant differences between groups in terms of smoking reduction or smoking cessation rates at 6 or 12 months. ^(^ 13 ^,^ 40 ^)^ The three prospective uncontrolled studies involved a small number of smokers unwilling to quit (one of the groups consisting of schizophrenic patients) and found smoking cessation rates of 22.5%, 14.3%, and 12.5% at 6 months, 12 months, and 24 months, respectively.^(^ 38 ^,^ 39 ^,^ 41 ^)^ In addition, an online survey of 5,000 individuals who had purchased a particular brand of ECs was conducted 7 months after the purchase and showed high rates of smoking reduction (66.8%) and smoking cessation (31.0%) at 6 months among the 222 questionnaire respondents.^(^ 54 ^)^ However, one limitation of that study was that the survey response rate was low, i.e., 4.5%; if the survey nonrespondents were to be considered smokers, the smoking cessation rate would be 1.4%.^(^ 54 ^)^

The studies described above show a low rate of smoking cessation with the use of ECs in a population relying on self-treatment and self-reporting smoking cessation. However, a survey of EC users showed frequent and prolonged EC use (approximately 20 times per day for more than 1 year), often in association with conventional cigarettes.^(^ 55 ^)^ In addition, some smokers with no intention to quit use ECs as a substitute for conventional cigarettes in places where smoking is prohibited. Therefore, EC use modulated by the need for nicotine can contribute to the maintenance of nicotine dependence. 

## Advertisement, impact on public health, and regulatory issues

Manufacturers of ECs have used aggressive advertising to encourage EC use. The main arguments used by the EC industry are the health benefits of ECs in comparison with conventional cigarettes, smoking reduction, smoking cessation, minimal passive exposure, and the possibility of using ECs in places where smoking is prohibited. ^(^ 55 ^)^ In 2012, a large tobacco company (Lorillard Tobacco Company) acquired an EC brand and began to run television and Internet advertisements starring celebrities and suggesting that the EC is glamorous and modern.^(^ 56 ^,^ 57 ^)^ These strategies have proven useful, given that EC use has increased. 

Professionals working to reduce tobacco consumption are increasingly concerned about the impact of ECs on public health. The reasons for this concern are as follows: the lack of data on the efficacy of ECs in smoking cessation; the potential to induce nicotine addiction in nonsmokers, especially children and adolescents; the simultaneous use of conventional cigarettes and ECs, reducing smoking cessation attempts; the possibility that ECs will undermine tobacco-free environments, making smoking acceptable; and exposure to a new form of pollution in places where smoking has been banned.^(^ 10 ^,^ 58 ^)^

In the USA, ECs have yet to be regulated as drugs or tobacco products.^(^ 10 ^)^ In the European Union and the UK, it has been proposed that ECs be regulated as medicinal products.^(^ 59 ^)^ Through regulation, ECs have been banned in Australia, Canada, Singapore, and Brazil because of the lack of data on their safety and efficacy.^(^ 12 ^,^ 16 ^)^

## How to counsel patients regarding EC use

**Chart 2:**
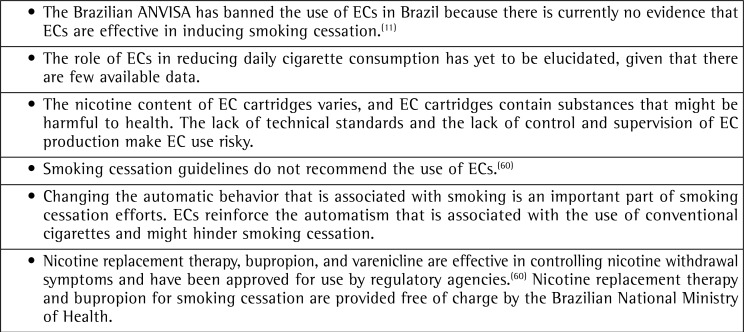
Counseling patients on the use of electronic cigarettes EC: electronic cigarette; and ANVISA: Agência Nacional de Vigilância Sanitária (National Health Surveillance Agency).

On the basis of the aforementioned information, pulmonologists can and should counsel patients seeking information on ECs. Chart 2 shows EC issues that can be addressed. It is possible that patients seeking information on ECs are motivated to quit smoking. Cognitive-behavioral therapy should be offered to all smokers. Smoking cessation guidelines^(^ 60 ^)^ contain scientifically proven information on how to help patients quit smoking. Nicotine withdrawal treatment is available in the public health system.^(^ 5 ^)^ For smokers with a high level of dependence, the combined use of medications to control withdrawal symptoms can increase treatment effectiveness. Nicotine replacement therapy, bupropion, and varenicline are treatment options approved by the Brazilian National Health Surveillance Agency. 

## Final considerations

The EC is an electronic nicotine delivery system that has gained popularity in recent years. However, EC sales are prohibited in Brazil. The dose of nicotine delivered and the contents of EC cartridges vary widely across EC brands. Short-term adverse health effects of EC use have been reported. The long-term toxicity of ECs has yet to be studied. Data on the efficacy of ECs in smoking cessation are scarce, and the role of ECs in inducing smoking cessation has yet to be confirmed. Prolonged use of ECs by smokers might perpetuate nicotine addiction, and EC use during adolescence might encourage smoking initiation. Therefore, a smoking cessation approach based on cognitive-behavioral therapy and the use of drugs approved by regulatory agencies in order to control nicotine withdrawal symptoms should be recommended for smokers who want to quit smoking.

## References

[1] World Health Organization (2011). [homepage on the Internet].

[2] Instituto Nacional de CâncerJosé Alencar Gomes da Silva www.inca.gov.br.

[3] Malta DC, Moura EC, Silva SA, Oliveira PP, Silva VL (2010). Prevalence of smoking among adults residing in the
Federal District of Brasília and in the state capitals of Brazil,
2008. J Bras Pneumol..

[4] World Health Organization [homepage on the Internet] http://whqlibdoc.who.int/publications/2003/9241591013.pdf.

[5] Portal de Saúde [homepage on the internet] http://dtr2001.saude.gov.br/sas/PORTARIAS/Port2004/GM/GM-1035.htm.

[6] Jarvis MJ, McIntiyre NC, Bates C, Foulds J (2002). Effectiveness of smoking cessation initiatives. Efforts
must take into account smokers' disillusionment with smoking and their delusions
about stopping. BMJ.

[7] Aveyard P, West R (2007). Managing smoking cessation. BMJ.

[8] Hughes JR, Keely J Naud S (2004). Shape of the relapse curve and long-term abstinence
among untreated smokers. Addiction.

[9] Cahn Z, Siegel M (2011). Electronic cigarettes as a harm reduction strategy for
tobacco control: a step forward or a repeat of past mistakes?. J Public Health Policy.

[10] Benowitz NL, Goniewicz ML (2013). The regulatory challenge of electronic
cigarettes. JAMA.

[11] Odum LE, O'Dell KA, Schepers JS (2012). Electronic cigarettes: do they have a role in smoking
cessation?. J Pharm Pract.

[12] Brasil. Ministério da Saúde. Agência Nacional de Vigilância
Sanitária (2009). Resolução de Diretoria Colegiada no. 46, de 28 de agosto de
2009. Proíbe a comercialização, a importação e a propaganda de quaisquer
dispositivos eletrônicos para fumar, conhecidos como cigarro eletrônico.

[13] Bullen C, Howe C, Laugesen M, McRobbie H, Parag V, Williman J (2013). Electronic cigarettes for smoking cessation: a
randomised controlled trial. Lancet.

[14] World Health Organization [homepage on the Internet] http://www.who.int/tobacco/communications/statements/eletronic_cigarettes/en/index.html.

[15] Goniewicz ML, Knysak J, Gawron M, Kosmider L, Sobczak A, Kurek J (2014). Levels of selected carcinogens and toxicants in vapour
from electronic cigarettes. Tob Control.

[16] World Health Organization Study Group on Tobacco Regulation (2009). TobReg scientific recommendation: devices designed for
the purpose of nicotine to the respiratory system in which tobacco is not
necessary for their operation. WHO Technical Report Series 955. Report on the scientific basis
of tobacco regulation: third report of a WHO study group.

[17] Westenberger BJ (2009). Evaluation of e-cigarettes. US Food and Drug Administration;
Center for Drug Evaluation and Research; Division of Pharmaceutical
Analysis.

[18] Bertholon JF, Becquemin MH, Annesi-Maesano I, Dautzenberg B (2013). Electronic cigarettes: a short review. Respiration.

[19] Farsalinos KE, Spyrou A, Tsimopoulou K, Stefopoulos C, Romagna G, Voudris V (2014). Nicotine absorption from electronic cigarette use:
comparison between first and new-generation devices. Sci Rep..

[20] Yamin CK, Bitton A, Bates DW (2010). E-cigarettes: a rapidly growing Internet
phenomenon. Ann Intern Med.

[21] Dockrell M, Indu SD, Lashkari HG, McNeill A (2010). "It sounds like the replacement I need to help me stop
smoking": Use and acceptability of "e-cigarettes" among UK smokers.

[22] Pepper JK, Brewer NT (2014). Electronic nicotine delivery system (electronic
cigarette) awareness, use, reactions and beliefs: a systematic
review. Tob Control.

[23] Regan AK, Promoff G, Dube SR, Arrazola R (2013). Electronic nicotine delivery systems: Adult use and
awareness of the 'e-cigarette' in the USA. Tob Control.

[24] Pearson JL, Richardson A, Niaura RS, Vallone DM, Abrams DB (2012). e-Cigarette awareness, use, and harm perceptions in US
adults. Am J Public Health..

[25] Dockrell M, Morrison R, Bauld L, McNeill A (2013). E-cigarettes: prevalence and attitudes in Great
Britain. Nicotine Tob Res.

[26] Adkison SE, O'Connor RJ, Bansal-Travers M, Hyland A, Borland R, Yong HH (2013). Electronic nicotine delivery systems: international
tobacco control four-country survey. Am J Prev Med.

[27] Etter JF, Bullen C (2011). Electronic cigarette: users profile, utilization,
satisfaction and perceived efficacy. Addiction.

[28] Etter JF (2010). Electronic cigarettes: a survey of users. BMC Public Health.

[29] Pepper JK, Reiter PL, McRee AL, Cameron LD, Gilkey MB, Brewer NT (2013). Adolescent males' awareness of and willingness to try
electronic cigarettes. J Adolesc Health.

[30] Cho JH, Shin E, Moon SS (2011). Electronic-cigarette smoking experience among
adolescents. J Adolesc Health.

[31] Centers for Disease Control and Prevention[homepage on the
Internet] (2012). http://www.cdc.gov/tobacco/data_statistics/surveys/nyts.

[32] Centers for Disease Control and Prevention (2013). Notes from the field: electronic cigarette use among
middle and high school students - United States, 2011-2012. MMWR Morb Mortal Wkly Rep.

[33] Sutfin EL, McCoy TP, Morrell HE, Hoeppner BB, Wolfson M (2013). Electronic cigarette use by college
students. Drug Alcohol Depend.

[34] Wieslander G, Norbäck D, Lindgren T (2001). Experimental exposure to propylene glycol mist in
aviation emergency training: acute ocular and respiratory effects. Occup Environ Med.

[35] World Health Organization [homepage on the Internet] http://www.who.int/mediacentre/news/releases/2008/pr34/en/index.html.

[36] Chen I (2013). FDA summary of adverse events on electronic
cigarettes. Nicotine Tob Res.

[37] Dawkins L, Corcoran O (2014). Acute electronic cigarette use: nicotine delivery and
subjective effects in regular users.

[38] Polosa R, Caponnetto P, Morjaria JB, Papale G, Campagna D, Russo C (2011). Effect of an electronic nicotine delivery device
(e-Cigarette) on smoking reduction and cessation: a prospective 6-month pilot
study. BMC Public Health.

[39] Caponnetto P, Auditore R, Russo C, Cappello GC, Polosa R (2013). Impact of an electronic cigarette on smoking reduction
and cessation in schizophrenic smokers: a prospective 12-month pilot
study. Int J Environ Res Public Health.

[40] Caponnetto P, Campagna D, Cibella F, Morjaria JB, Caruso M, Russo C, Polosa R (2013). EffiCiency and Safety of an eLectronic cigAreTte (ECLAT)
as tobacco cigarettes substitute: a prospective 12-month randomized control design
study. PLoS ONE.

[41] Polosa R, Morjaria JB, Caponnetto P, Campagna D, Russo C, Alamo A (2014). Effectiveness and tolerability of electronic cigarette
in real-life: a 24-month prospective observational study. Intern Emerg Med.

[42] Flouris AD, Poulianiti KP, Chorti MS, Jamurtas AZ, Kouretas D, Owolabi EO (2012). Acute effects of electronic and tobacco cigarette
smoking on complete blood count. Food Chem Toxicol.

[43] Farsalinos KE, Romagna G, Allifranchini E, Ripamonti E, Bocchietto E, Todeschi S (2013). Comparison of the cytotoxic potential of cigarette smoke
and electronic cigarette vapour extract on cultured myocardial
cells. Int J Environ Res Public Health.

[44] Flouris AD, Chorti MS, Poulianiti KP, Jamurtas AZ, Kostikas K, Tzatzarakis MN (2013). Acute impact of active and passive electronic cigarette
smoking on serum cotinine and lung function. Inhal Toxicol.

[45] Vardavas CI, Anagnostopoulos N, Kougias M, Evangelopoulou V, Connolly GN, Behrakis PK (2012). Short-term pulmonary effects of using an electronic
cigarette: impact on respiratory flow resistance, impedance, and exhaled nitric
oxide. Chest.

[46] Vink GR, Arets HG, van der Laag J, van der Ent CK (2003). Impulse oscillometry: a measure for airway
obstruction. Pediatr Pulmonol..

[47] American Thoracic Society Workshop (2006). ATS Workshop Proceedings: Exhaled nitric oxide and
nitric oxide oxidative metabolism in exhaled breath condensate: Executive
summary. Am J Respir Crit Care Med.

[48] Schober W, Szendrei K, Matzen W, Osiander-Fuchs H, Heitmann D, Schettgen T (2014). Use of electronic cigarettes (e cigarettes) impairs
indoor air quality and increases FeNO levels of e-cigarette
consumers. Int J Hyg Environ Health.

[49] Vansickel AR, Cobb CO, Weaver MF, Eissenberg TE (2010). A clinical laboratory model for evaluating the acute
effects of electronic "cigarettes": nicotine delivery profile and cardiovascular
and subjective effects. Cancer Epidemiol Biomarkers Prev.

[50] Eissenberg T (2010). Electronic nicotine delivery devices: ineffective
nicotine delivery and craving suppression after acute
administration. Tob Control.

[51] Bullen C, McRobbie H, Thornley S, Glover M, Lin R, Laugesen M (2010). Effect of an electronic nicotine delivery device (e
cigarette) on desire to smoke and withdrawal, user preferences and nicotine
delivery: randomised cross-over trial. Tob Control.

[52] Vansickel A, Eissenberg T (2013). Electronic cigarettes: effective nicotine delivery after
acute administration. Nicotine Tob Res.

[53] Dawkins L, Turner J, Hasna S, Soar K (2012). The electronic-cigarette: Effects on desire to smoke,
withdrawal symptoms and cognition. Addict Behav.

[54] Siegel MB, Tanwar KL, Wood KS (2011). Electronic cigarettes as a smoking cessation tool:
Results from an online survey. Am J Prev Med.

[55] Foulds J, Veldheer S, Berg A (2011). Electronic cigarettes (e-cigs): views of aficionados and
clinical/public health perspectives. Int J Clin Pract.

[56] Noel JK, Rees VW, Connolly GN (2011). Electronic cigarettes: a new "tobacco"
industry?. Tob Control.

[57] YouTube. [homepage on the Internet] (2012). http://www.youtube.com/watch?v=9pxuBgfbid0.

[58] Britton J (2013). Electronic cigarretes. Thorax.

[59] Hajek P (2013). Electronic cigarettes for smoking
cessation. Lancet.

[60] Reichert J, Araújo AJ, Gonçalves CM, Godoy I, Chatkin JM, Sales MP (2008). Smoking cessation guidelines--2008. J Bras Pneumol.

