# Supplementary material for: The electronic cigarette: the new cigarette of the 21st century?
Source: J Bras Pneumol. 2014 Sep-Oct;40(5):564–72. doi: 10.1590/S1806-37132014000500013 (PMC4263338; doi:10.1590/S1806-37132014000500013)
Supplement: Translation

# Translation: Cigarro eletrônico: o novo cigarro do século 21?*

**Authors:** Marli Maria Knorst, Igor Gorski Benedetto, Mariana Costa Hoffmeister, Marcelo Basso Gazzana

**Affiliations:** Faculdade de Medicina, Universidade Federal do Rio Grande do Sul; e Médica. Serviço de Pneumologia, Hospital de Clínicas de Porto Alegre, Porto Alegre (RS) Brasil; Programa de Pós-Graduação em Ciências Pneumológicas, Universidade Federal do Rio Grande do Sul, Porto Alegre (RS) Brasil; Faculdade de Medicina, Universidade Federal do Rio Grande do Sul, Porto Alegre (RS) Brasil; Serviço de Pneumologia, Hospital de Clínicas de Porto Alegre; e Serviço de Pneumologia e Cirurgia Torácica, Hospital Moinhos de Vento, Porto Alegre (RS) Brasil

**Keywords:** Hábito de Fumar, Produtos do Tabaco, Nicotina

## Abstract

O cigarro eletrônico é um sistema eletrônico de liberação de nicotina que está gerando controvérsias, tanto entre a população quanto entre profissionais da saúde. O uso crescente do cigarro eletrônico é observado em tabagistas de diversos países, tanto para auxiliar na cessação do tabagismo quanto como substituto do cigarro convencional. Dados sobre a segurança do uso do cigarro eletrônico são limitados. Do mesmo modo, até o momento, não há evidências de que o cigarro eletrônico seja efetivo para tratar a adição à nicotina. Usuários relataram usar o cigarro eletrônico por mais de um ano, frequentemente combinado com o cigarro convencional, prolongando assim a dependência de nicotina. Ainda, o uso crescente do cigarro eletrônico por adolescentes gera preocupação. Neste artigo é feita uma descrição do cigarro eletrônico e de seus constituintes, assim como são revistos os dados disponíveis sobre segurança, impacto na iniciação e na cessação do tabagismo, e questões relacionadas à regulação do uso do cigarro eletrônico.

## Introdução

O tabagismo é um importante problema de saúde pública no mundo e é considerado pela Organização Mundial da Saúde (OMS) como uma das principais causas de morte passíveis de prevenção.^(^ 1 ^)^ No Brasil ocorrem cerca de 220 mil mortes/ano relacionadas ao uso do tabaco. ^(^ 2 ^)^ Apesar disso, na população adulta brasileira, 16,1% são tabagistas, sendo 17 milhões de homens e 12,5 milhões de mulheres.^(3) ^A preocupação com a morbidade e a mortalidade associadas ao tabagismo desencadeou uma iniciativa da OMS - a Convenção Quadro para o Controle do Tabaco, implementada em 27 de fevereiro de 2005 e ratificada por 177 países, dentre os quais o Brasil.^(^ 4 ^)^ Entre as diretrizes dessa convenção estão a promoção de ambientes livres de fumo de tabaco e a implantação de projetos para a cessação do tabagismo. O Ministério da Saúde do Brasil, em concordância com a convenção quadro da OMS, estruturou e difundiu na rede pública do Sistema Único de Saúde um programa de abordagem do tabagismo baseado em terapias cognitivo-comportamental e medicamentosa.^(^ 5 ^)^

Estudos realizados com tabagistas mostram que, se pudessem voltar atrás no tempo, muitos não teriam iniciado a fumar^(^ 6 ^)^ e que 60-70% desses queriam parar de fumar.^(^ 7 ^)^ Entretanto, sem ajuda, a maioria das pessoas que tenta cessar o uso do cigarro recai, e somente 4% permanecem abstinentes em um ano.^(^ 8 ^)^ Um dos fatores mais importantes que dificultam a cessação do tabagismo é a dependência de nicotina. Nesse contexto, o cigarro eletrônico (CE, também referido como *e-cigarette* em inglês) surgiu como uma forma de reposição de nicotina. Ele foi desenvolvido pelo farmacêutico chinês Hon Lik e patenteado em 2003.^(^ 9 ^)^ Embora com dados precários sobre sua eficácia e segurança de uso, o CE vem tendo sua comercialização disseminada através da Internet, bem como diretamente ao consumidor em vários países.

Atualmente, mais de 2.500 marcas de CE são vendidas no mundo.^(^ 10 ^)^ Diversas dessas marcas foram adquiridas pela indústria do tabaco. O custo do CE nos EUA varia de U$ 29,95 a U$ 149,95 e, do cartucho, de U$ 9,95 a U$ 19,95. ^(^ 11 ^)^ No Brasil, a comercialização, a importação e a propaganda do CE foram proibidas pela Agência Nacional de Vigilância Sanitária em 2009.^(^ 12 ^)^ Apesar disso, os tabagistas têm acesso ao dispositivo e com frequência procuram o pneumologista para questionar sobre a indicação e a eficácia do mesmo. O objetivo do presente estudo foi descrever o CE e revisar as evidências disponíveis sobre o papel do CE na cessação do tabagismo, seu impacto na iniciação do tabagismo, a segurança no uso do CE, assim como aspectos éticos e regulatórios relacionados com esse tipo de dispositivo de reposição de nicotina.

## Características do CE

O CE é um dispositivo eletrônico que fornece aos usuários doses de nicotina e outros aditivos em aerossol. São três os componentes principais do CE: uma bateria, um atomizador e um cartucho contendo nicotina (Figura 1). Em alguns países é comercializada uma versão de CE sem nicotina. ^(^ 13 ^)^ Alguns CEs têm um indicador luminoso na ponta que acende quando o usuário usa o aparelho, lembrando-o assim que o cigarro está aceso. A maioria dos sistemas eletrônicos de liberação de nicotina imita as formas tradicionais de utilização do tabaco, isto é, o cigarro, o charuto ou o cachimbo; menos frequentemente, o CE tem a forma de um objeto de uso diário, como uma caneta ou um *pen drive*, sendo usado principalmente por indivíduos que querem fumar sem chamar a atenção.^(^ 14 ^)^

**Figura 1:** Componentes do cigarro eletrônico. LED: light-emitting diode (díodo emissor de luz).

A constituição do cartucho varia de acordo com a marca comercial e geralmente possui nicotina e um componente para produzir o aerossol (por exemplo, propilenoglicol ou glicerol diluído em água). O nível de nicotina no cartucho pode variar e não corresponder à concentração descrita pelo fabricante.^(^ 15 ^,^ 16 ^)^ Algumas marcas de CE podem conter substâncias que modificam o sabor, como extrato de frutas, baunilha, menta, café ou chocolate, tornando o CE mais atrativo principalmente para adolescentes. Diversas substâncias potencialmente danosas, como formaldeído, acetaldeído, acroleína, compostos orgânicos voláteis, metais pesados e nitrosaminas derivadas do tabaco, foram identificadas nos cartuchos de nicotina.^(^ 15 ^,^ 17 ^)^

Quando o usuário aspira o dispositivo, um sensor detecta o fluxo de ar e aquece o líquido do cartucho, causando a evaporação. O vapor libera a nicotina para o usuário, e uma parte do vapor pode ser liberada no ar ambiente quando o usuário exala. A temperatura do vapor atinge 40-65°C. De acordo com informações dos fabricantes, um cartucho pode gerar de 10 a 250 jatos, o que poderia corresponder, dependendo da marca, a 5-30 cigarros.^(^ 18 ^)^ Recentemente foram desenvolvidos CEs de segunda e terceira geração, com baterias e vaporizadores mais potentes, capazes de liberar doses maiores de nicotina, aumentando assim o risco de dependência.^(^ 19 ^)^

## Prevalência do uso do CE em adultos

Embora haja falta de evidências demonstrando a eficácia do CE na cessação do tabagismo, cresce o número de interessados sobre o assunto, assim como o número de consumidores no mundo, sendo a maioria constituída por adultos tabagistas.^(^ 20 ^-^ 22 ^)^ Os usuários do CE se autodenominam *vapers*. Nos EUA, uma pesquisa com mais de 10.000 indivíduos adultos mostrou que o conhecimento da existência do CE duplicou entre 2009 e 2010 (de 16,4% para 32,2%), e o uso do CE praticamente quadruplicou, passando de 0,6% em 2009 para 2,7% em 2010.^(^ 23 ^)^ Entre tabagistas ativos, 11,4% referiram terem usado o CE, e 4,1% relataram seu uso nos últimos 30 dias.^(^ 24 ^)^ Na Grã-Bretanha, a taxa de usuários regulares do CE aumentou de 2,7% em 2010 para 6,7% em 2012.^(^ 25 ^)^ Dados obtidos de 5.939 indivíduos em quatro países (EUA, Reino Unido, Canadá e Austrália) entre 2010 e 2011 mostraram que cerca da metade dos indivíduos entrevistados (46,6%) tinham conhecimento da existência do CE. Entretanto, a taxa de indivíduos cientes da existência do CE variou significativamente entre os países, sendo maior nos EUA (73,4%) e no Reino Unido (54,4%), onde o acesso ao CE é permitido e menor no Canadá (39,5%) e Austrália (20,0%), onde o CE foi banido. A taxa de experimentação foi de 7,6% (16,3% entre os que conheciam o CE) e a de uso atual foi de 3%; a proporção de consumidores atuais do CE não variou entre os diferentes países (p = 0,114).^(^ 26 ^)^

Um fórum on-line dedicado à cessação do tabagismo e ao CE, realizado na Inglaterra e na França em 2010, reuniu 3.587 participantes (70% ex-tabagistas, 61% homens, com média de idade de 41 anos). O CE com nicotina foi usado por 97% dos participantes, sendo usado por cerca de cinco meses entre ex-tabagistas. A maioria referiu que o CE os ajudou a parar de fumar ou a reduzir o consumo (96%). Dentre as razões para o uso do CE, foram relatados a percepção de que o CE seria menos tóxico que o cigarro (87%), a redução da fissura pelo tabaco (79%), a redução dos sintomas de abstinência (77%), o custo inferior ao tabaco (57%) e o controle de situações nas quais era proibido fumar (39%).^(^ 27 ^)^ Uma das limitações do estudo foi a seleção da amostra por conveniência. Outra pesquisa on-line realizada com 81 tabagistas mostrou o padrão de consumo de usuários regulares do CE. A mediana do tempo de uso do CE foi de 100 dias, e a do número de jatos/dia foi de 175.^(^ 28 ^)^

Uma revisão sistemática recente que incluiu 49 estudos mostrou que o conhecimento da existência do CE passou de 16% em 2009 para 58% em 2011 e que o uso do CE aumentou de 1% para 6% no mesmo período.^(^ 22 ^)^

## Exposição de adolescentes ao CE

O CE é conhecido e é acessível para uso de crianças e adolescentes em diversos países. Uma pesquisa on-line com 228 adolescentes americanos do sexo masculino mostrou que 67% conheciam o CE, embora menos de 1% afirmassem tê-lo experimentado.^(^ 29 ^)^ Em um estudo com 444 adolescentes coreanos, 10,2% referiram ter visto ou ouvido sobre o CE, e 0,5% relataram tê-lo usado. A rota de contato com o CE foi a Internet, em 46% dos casos; amigos, em 27,9%; televisão, em 11,0%; livros, em 9,3%; e outros, em 5,4%. A probabilidade de usar CE foi 6,3 vezes maior entre adolescentes meninos que entre meninas e 3,4 vezes maior nos adolescentes com tabagistas na família que naqueles sem tabagistas na família.^(^ 30 ^)^

A prevalência do uso de CE e de cigarros convencionais em alunos do 6° ao 12° ano entre os anos de 2011 e 2012 foi estudada nos EUA através de uma pesquisa transversal (*National Youth Tobacco Survey*).^(^ 31 ^)^ Os resultados mostraram que tanto a experimentação como o uso recente de CE praticamente duplicou em 1 ano. O uso de CE aumentou de 3,3% para 6,8% (p < 0,05), o uso de cigarro convencional aumentou de 1,1% para 2,1% (p < 0,05), enquanto o uso de ambos foi de 0,8% para 1,6% (p = 0,05) no período de estudo. Não houve diferenças nesses incrementos quando foram comparados os grupos de alunos do 6° ao 8° ano e do 9° ao 12° ano de ensino. O estudo mostrou ainda que, em 2012, entre os experimentadores de CEs, 9,3% referiram nunca fumar cigarros convencionais, e, entre os usuários regulares do CE, 76% referiram fumar regularmente cigarros tradicionais.^(^ 32 ^)^

Outro estudo realizado em oito escolas da Carolina do Norte (EUA) com 4.444 adolescentes entre 11 e 19 anos relatou o uso de CE em 4,9% dos estudantes, sendo que 1,5% referiam seu uso no último mês. Embora o uso do CE fosse mais comum entre os adolescentes que já fumassem, 12% dos consumidores de CE nunca haviam usado o cigarro tradicional.^(^ 33 ^)^

Tanto a propaganda na Internet como a comercialização on-line do CE, mesmo nos países nos quais o produto foi banido, podem estimular o consumo e permitem que os adolescentes tenham acesso ao produto. Adicionalmente, os dados dos estudos acima citados^(^ 29 ^-^ 33 ^)^ sugerem que a experimentação do CE poderia induzir o uso continuado de cigarros tradicionais nessa fase crítica da vida. Assim, ações que reduzam o apelo do consumo de CE e que proíbam sua venda a adolescentes são fundamentais para minimizar o risco do consumo do tabaco e/ou do CE. 

## Segurança do uso de CE

A segurança dos sistemas eletrônicos de liberação da nicotina não foi cientificamente demonstrada, e o risco potencial para a saúde do usuário ainda permanece indeterminado. A maior parte dos problemas de segurança relacionada ao CE é devida à ausência de regulação adequada e de inconsistências no seu controle de qualidade. A falta de regulação e de fiscalização resulta numa variabilidade grande na qualidade dos dispositivos, na quantidade de nicotina dispensada e nos demais constituintes do cartucho entre as diferentes marcas.^(^ 15 ^)^ Assim, o consumidor não tem como saber a real constituição do produto que está usando.

Efeitos adversos do CE podem resultar do conteúdo variável de nicotina no cartucho. Segundo a informação dos fabricantes, a quantidade de nicotina por cartucho pode variar de 6-24 mg; entretanto, algumas vezes, doses de até 100 mg foram detectadas por cartucho. Portanto, o risco de intoxicação deve ser considerado. A nicotina, ao ser inalada, ingerida ou ao entrar em contato com a pele, pode ser perigosa para a saúde de grupos vulneráveis, como crianças, jovens, gestantes, nutrizes, portadores de doenças cardíacas e idosos. A nicotina em grandes quantidades (0,5-1,0 mg por kg de peso corporal) pode ser letal, sendo recomendado que o CE, seu cartucho e acessórios de recarga sejam mantidos fora do alcance de crianças.^(^ 14 ^,^ 16 ^)^

Os riscos à saúde podem também estar associados com as diferentes substâncias encontradas nos cartuchos substituíveis do CE. Uma delas é o propilenoglicol, que é a substância na qual a nicotina fica em suspensão e serve para gerar o vapor. Dados sobre os efeitos nocivos da inalação do propilenoglicol são escassos. Irritação ocular e das vias aéreas superiores, assim como a presença de tosse e obstrução leve das vias aéreas, foram descritas após a exposição curta à névoa de propilenoglicol criada por um gerador de fumaça artificial em alguns indivíduos não asmáticos.^(^ 34 ^)^

Outras substâncias potencialmente nocivas, incluindo irritantes e toxinas, como dietilenoglicol, formaldeído, acetaldeído e acroleína, também foram detectadas em algumas marcas de CE.^(^ 17 ^)^ Nitrosaminas, que são carcinógenos bem reconhecidos,^(^ 35 ^)^ assim como impurezas específicas relacionadas ao tabaco, foram encontradas em concentrações baixas em duas marcas comerciais de CE.^(^ 17 ^)^ O CE pode conter substâncias aromatizantes, que são acrescentadas ao cartucho para tornar o CE mais palatável. Embora essas substâncias sejam condimentos alimentares usados na rotina, os efeitos da inalação das mesmas não são conhecidos. 

Nos EUA, a *Food and Drug Administration* recebeu, até o primeiro trimestre de 2012, 49 relatos de eventos adversos com o uso do CE. Desses, 8 foram considerados eventos graves, incluindo pneumonia e dor torácica, e os demais foram caracterizados como leves, e incluíram cefaleia e tosse.^(^ 36 ^)^ Um estudo que avaliou os efeitos adversos agudos após 2,5 h de uso do CE relatou cefaleia leve, irritação de boca e garganta, salivação, sudorese, fraqueza, palpitações, náuseas, vômitos e diarreia entre os principais sintomas. Entretanto, tais efeitos foram leves.^(^ 37 ^)^ Em três estudos prospectivos nos quais tabagistas usaram o CE por 6 ou 12 meses, não foram observados eventos adversos graves, e as principais queixas relatadas foram tosse, cefaleia e irritação na boca e garganta. Os sintomas desapareceram ou reduziram de intensidade com o uso.^(^ 38 ^-^ 41 ^)^

Estudos in vivo e in vitro avaliaram o impacto do vapor do CE sobre as células sanguíneas e o efeito citotóxico sobre as células miocárdicas. Num estudo in vivo, o vapor do CE, tanto no seu uso ativo como na exposição passiva por 30 min, não alterou as contagens de leucócitos, linfócitos e granulócitos.^(^ 42 ^)^ Outro estudo in vitro avaliou o potencial citotóxico do vapor proveniente de 20 marcas de CE para células miocárdicas em cultura.^(^ 43 ^)^ Os autores demonstraram que algumas amostras apresentavam efeitos citotóxicos para células miocárdicas, embora a citotoxicidade do vapor do CE fosse menor que a da fumaça do cigarro tradicional.^(^ 43 ^)^

Os efeitos do CE sobre a função pulmonar foram estudados apenas após sua exposição aguda. O uso do CE por tabagistas durante alguns minutos, assim como a exposição passiva de não tabagistas ao CE durante 1 h, não apresentou efeitos sobre o VEF_1_.^(^ 44 ^)^ Por outro lado, o uso do CE durante 5 min aumentou a resistência nas vias aéreas e reduziu a fração de óxido nítrico exalado em tabagistas adultos sem outras doenças.^(45) ^O aumento da resistência nas vias aéreas pode preceder as alterações no PFE e no VEF_1_ na obstrução ao fluxo aéreo induzida experimentalmente.^(^ 46 ^)^ Reconhecidamente, o óxido nítrico desempenha um papel na fisiopatologia das doenças das vias aéreas associadas ao tabagismo, está relacionado à inflamação eosinofílica e à hiperreatividade brônquica e é um marcador de estresse oxidativo.^(^ 47 ^)^ Observados em conjunto, os achados acima sugerem que alterações pulmonares são induzidas pelo uso do CE em curto prazo. Efeitos sobre a função pulmonar em longo prazo não foram estudados.

Como o CE não gera a fumaça que está associada com a combustão do tabaco, a sua utilização é geralmente considerada mais segura para os consumidores do que o tabaco. Essa "segurança relativa" pode ser atraente para os consumidores; no entanto, os produtos químicos utilizados no CE não foram totalmente revelados, e dados sobre a poluição ambiental gerada pelo uso do CE em ambientes fechados são escassos. Um estudo avaliou a qualidade do ar num ambiente com 9 indivíduos usando o CE. Os resultados mostraram quantidades substanciais de 1,2-propanodiol, glicerina e nicotina, altas concentrações de material particulado com 2,5 mm, um incremento de 20% nos níveis de hidrocarbonetos aromáticos policíclicos e um aumento nos níveis de alumínio e de material particulado total. A concentração de óxido nítrico no ar exalado aumentou em 7 dos 9 indivíduos.^(^ 48 ^)^ Adicionalmente, dados de segurança associados ao uso do CE em longo prazo também não estão disponíveis.^(^ 14 ^)^

## Eficácia do CE na cessação do tabagismo

Os dados disponíveis sobre o CE até o momento são muito limitados. Segundo a OMS, não há comprovação científica para o uso de CEs como substitutivo ou auxiliar na cessação do consumo do tabaco. Do mesmo modo, a forma de administração da nicotina diretamente para o pulmão difere das formas liberadas de reposição da nicotina (adesivo, goma e pastilhas) e precisa ser estudada.^(^ 14 ^)^

A comparação de CEs de diferentes marcas mostrou concentrações variáveis de nicotina nos cartuchos, assim como inconsistências no conteúdo dos jatos e na liberação da nicotina. Após uso do CE, os níveis plasmáticos de nicotina permaneceram inalterados em todos os pacientes em dois estudos,^(^ 49 ^,^ 50 ^)^ e em cerca de um terço dos casos em outro estudo.^(^ 51 ^)^ Entretanto, em usuários habituais de CEs, os níveis plasmáticos de nicotina podem aumentar,^(^ 52 ^)^ embora uma grande variabilidade individual nos níveis séricos de cotinina tenha sido observada.^(^ 37 ^)^

Diversos estudos avaliaram o impacto do CE sobre o desejo de fumar e sobre a fissura. Num estudo randomizado e cruzado patrocinado por um fabricante de CE, o dispositivo contendo 16 mg de nicotina foi mais efetivo que o placebo para aliviar os sintomas de abstinência pela manhã em 40 indivíduos que ficaram sem fumar durante a noite. Os efeitos do CE sobre os sintomas de abstinência da nicotina foram comparáveis ao do inalador de nicotina, porém inferiores ao do próprio cigarro.^(^ 51 ^)^ Um grupo de autores^(^ 53 ^)^ estudou os efeitos sobre o desejo de fumar, os sintomas de abstinência e a cognição em 86 pacientes randomizados em três grupos: a) uso de CE (18 mg nicotina); b) uso do mesmo dispositivo sem nicotina; ou c) apenas segurar o CE. Em 20 min após seu uso, foi observada uma redução no desejo de fumar e nos sintomas de abstinência à nicotina com o uso do CE com nicotina e placebo em comparação com o grupo que apenas segurou o CE. A redução do desejo de fumar com o CE com nicotina, em comparação com o do grupo placebo, foi significativamente diferente apenas nos homens. Entretanto, o grupo que recebeu nicotina apresentou resultados significativamente melhores em testes de memória. Em outro estudo cruzado, foram comparados, em um protocolo de exposição aguda com 10 tragadas sequenciais a intervalos de 30 s entre as mesmas, duas marcas de CE com 16 mg de nicotina, um cigarro habitualmente usado pelo indivíduo e um cigarro não aceso (placebo). A redução da fissura em relação ao uso de placebo foi observada com uma das duas marcas de CE; entretanto, o efeito foi inferior ao do cigarro tradicional.^(^ 50 ^)^

Poucos estudos avaliaram os efeitos do CE na redução e na cessação do tabagismo num período de 6 a 24 meses. Dois ensaios clínicos randomizados controlados e três estudos prospectivos tipo antes e depois estão descritos no Quadro 1. Um dos ensaios clínicos^(^ 13 ^)^ comparou o uso de CE (16 mg de nicotina) com o de adesivos de nicotina (21 mg) e de CE sem nicotina, enquanto o outro^(^ 40 ^)^ comparou o uso de CE (7,2 mg de nicotina) com o de CE sem nicotina. Ambos os ensaios, administrados por 12 semanas, não encontraram diferenças significativas entre os grupos na redução do número de cigarros ou nas taxas de cessação em 6 ou 12 meses.^(^ 13 ^,^ 40 ^)^ Os três estudos prospectivos não controlados, realizados com um pequeno número de tabagistas que não pensavam em parar de fumar (um dos grupos era constituído por pacientes esquizofrênicos), descreveram taxas de cessação em 6, 12 e 24 meses de 22,5%, 14,3% e 12,5%, respectivamente. ^(^ 38 ^,^ 39 ^,^ 41 ^)^ Ainda, uma pesquisa on-line realizada com 5.000 indivíduos que adquiriram o CE de uma determinada marca, 7 meses após a aquisição, mostrou altas taxas de redução do consumo (66,8%) e de cessação (31,0%) em 6 meses entre os 222 respondedores do questionário.^(^ 54 ^)^ Entretanto, aquele estudo apresentou a limitação de que somente 4,5% dos indivíduos convidados responderam o questionário; se os indivíduos que não responderam o questionário fossem considerados como fumantes, a taxa de cessação seria de 1,4%.^(^ 54 ^)^

**Quadro 1:** Estudos clínicos que avaliaram os efeitos do cigarro eletrônico na redução e na cessação do tabagismo ECR: ensaio clínico randomizado; CO: monóxido de carbono; CE: cigarro eletrônico; e G1, G2 e G3: grupos 1, 2 e 3.

Os estudos descritos acima evidenciam uma baixa taxa de abandono do tabagismo com a utilização do CE em uma população que se autotrata e faz autorrelato da cessação do tabagismo. Por outro lado, a pesquisa com usuários descreveu o uso frequente e prolongado do CE, cerca de 20 vezes por dia por mais de 1 ano, muitas vezes associado ao uso do cigarro convencional.^(^ 55 ^)^ Ainda, alguns tabagistas, sem a mínima intenção de parar de fumar, utilizam o CE como substituto do cigarro convencional em locais onde é proibido fumar. Assim, o uso do CE modulado pela necessidade do consumo de nicotina pode contribuir para a manutenção da dependência de nicotina.

## Propaganda, impacto sobre a saúde pública e aspectos regulatórios

As companhias que produzem CEs têm usado propagandas agressivas para estimular o seu uso. Os principais argumentos usados pela indústria são os benefícios à saúde em comparação com o cigarro tradicional, a redução do consumo de cigarros, a cessação do tabagismo, a minimização da exposição passiva e a possibilidade de uso dos CEs em locais onde o fumo é proibido.^(^ 55 ^)^ Em 2012, uma grande corporação de tabaco (*Lorillard Tobacco Company*) adquiriu uma marca de CE e passou a divulgar comerciais com celebridades na televisão e na Internet, sugerindo que o CE é glamoroso e moderno.^(^ 56 ^,^ 57 ^)^ Essas estratégias se mostraram supostamente úteis, uma vez que o consumo do CE aumentou.

Por outro lado, cresce a preocupação de profissionais que trabalham para reduzir o consumo do tabaco sobre o impacto da utilização dos CEs no contexto de saúde pública. As razões para essa preocupação são as seguintes: a ausência de dados de eficácia na cessação do tabagismo; o potencial de induzir dependência à nicotina em não tabagistas, principalmente em crianças e adolescentes; a promoção do uso simultâneo do cigarro tradicional e do CE, reduzindo as tentativas de cessação; a sabotagem aos ambientes livres do tabaco através do uso de CEs, tornando o hábito de fumar aceitável; e a exposição a uma nova forma de poluição nos locais onde o tabagismo havia sido banido.^(^ 10 ^,^ 58 ^)^

Os CEs não têm regulamentação nos EUA nem como medicamento nem como produto derivado do tabaco.^(^ 10 ^)^ A União Europeia e o Reino Unido estão propondo regulamentar os CEs como um produto medicinal.^(^ 59 ^)^ Por causa da falta de dados sobre sua segurança e eficácia, o uso dos CEs foi banido na Austrália, no Canadá, em Singapura e no Brasil, através de regulação.^(^ 12 ^,^ 16 ^)^

## Como orientar o paciente sobre CEs

Com base nos conhecimentos acima, o médico pneumologista pode e deve orientar o paciente que busca informações sobre CEs. O Quadro 2 lista alguns aspectos dos CEs que podem ser abordados. A busca de informações pode significar que o paciente está motivado para parar de fumar. A terapia cognitivo-comportamental deve ser oferecida a todos os pacientes tabagistas. As diretrizes para cessação do tabagismo^(^ 60 ^)^ contêm informações cientificamente comprovadas de como ajudar o paciente a parar de fumar. O tratamento para os sintomas de abstinência da nicotina é disponibilizado na rede pública de saúde.^(^ 5 ^)^ Para fumantes com alto grau de dependência, o uso combinado de medicações para controlar os sintomas de abstinência pode aumentar a efetividade do tratamento. A reposição de nicotina, a bupropiona e a vareniclina são alternativas de tratamento aprovadas pela Agência Nacional de Vigilância Sanitária.

**Quadro 2:** Orientações para pacientes sobre o cigarro eletrônico. CE: cigarro eletrônico; e ANVISA: Agência Nacional de Vigilância Sanitária.

## Considerações finais

O CE é um sistema eletrônico de liberação de nicotina que ganhou popularidade nos últimos anos. Entretanto, a comercialização do mesmo é proibida no Brasil. Há uma grande variabilidade nas doses liberadas de nicotina e nos constituintes dos cartuchos entre as diferentes marcas de CE. Efeitos adversos à saúde em curto prazo foram descritos. A toxicidade do CE em longo prazo não foi estudada. Dados avaliando a eficácia do CE na cessação do tabagismo são escassos, e o seu papel para induzir a cessação do tabagismo não foi comprovado. A utilização prolongada do CE por tabagistas pode perpetuar a dependência à nicotina, e o seu uso durante a adolescência pode estimular a iniciação do tabagismo. Portanto, uma abordagem do tabagismo que contemple a terapia cognitivo-comportamental, assim como a utilização de medicamentos aprovados pelas agências regulatórias para o controle dos sintomas de abstinência à nicotina, deve ser oferecida aos tabagistas que querem parar de fumar.
